# Spontaneous aggregation of the anti-viral MAVS protein in systemic lupus erythematosus: a possible cause of excessive type I interferon production

**DOI:** 10.1186/ar3965

**Published:** 2012-09-27

**Authors:** PL Cohen, B Hilliard, W-H Shao

**Affiliations:** 1 Temple University School of Medicine, Philadelphia, PA, USA

## Rationale

Patients with systemic lupus erythematosus (SLE) often have evidence of excessive type I interferon production, with increased interferon levels and activation of interferon-inducible genes (interferon signature). The mitochondrial adaptor protein MAVS (also known as IPS1, VISA or CARDIF) is a key intermediary in the RIG-I pathway, where viral RNA triggers a conformational change in RIG-I, leading to MAVS activation and activation of IKK and TBK1, with subsequent interferon production driven by IRF-3 and NFκB activation and translocation. A recent report using *in vitro *methods demonstrated that MAVS may form large prion-like aggregates which might stimulate IFN-I activation in a potent and prolonged fashion [1]. We wondered whether such aggregates might be detectable *ex vivo *in SLE patients, and whether they might play a role in the sustained increased production of IFN-I. The aim was to determine whether aggregated MAVS protein is present in blood cells from SLE patients.

## Methods

Peripheral blood mononuclear cells (PBMC) were isolated from 17 patients fulfilling ACR criteria for SLE, and from nine controls. Thirty million PBMC were lysed and pellets loaded onto semi-denaturing detergent vertical 1.5% agarose gels. After electrophoresis, the proteins were transferred to Immobilon membranes for immunoblotting with anti-MAVS antibody or anti-β-actin.

## Results

**Figure 1:**
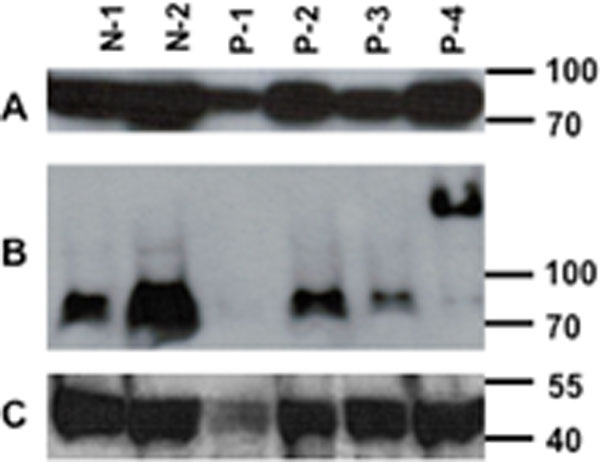


Six of 17 SLE patients showed clear MAVS aggregation, with essentially all of their MAVS protein in a high-molecular-weight form. None of nine controls had abnormal MAVS. Three of the four SLE patients had nephritis and the fourth had lung involvement. SLEDAI scores of MAVS-aggregate positive SLE patients did not differ from patients with normal molecular weight MAVS. Patient 4 (P-4) shows the aggregated MAVS phenotype in the western blot (Figure 1). MAVS immunoblotting is shown in panel B and actin immunoblotting in panel C. N-1 and N-2 are normal controls. P-1 has less protein loaded and no MAVS band is discernible.

## Conclusion

This is the first report of aggregated MAVS in human cells. The relationship of this abnormality to disease needs further investigation, but suggests the possibility that prolonged and increased IFN-I production could result from MAVS aggregation, with the formation of poorly degradable prion-like protein that could signal IFN-I production for prolonged periods.
